# A Multiscale Modeling Approach for the Prediction of the Mechanical Properties of C/SiC Composites Fabricated by the CVI Process

**DOI:** 10.3390/ma19030623

**Published:** 2026-02-06

**Authors:** Taegeon Kil, Yongyoon Cho, Jin-Ho Bae, Ji Eun Lee, Jong Sung Won, Man Young Lee, Hyung Ik Lee

**Affiliations:** 1 Agency for Defense Development (ADD), Yuseong, P.O. Box 35, Daejeon 34186, Republic of Korea; xorjs95175@gmail.com (T.K.); yycho@add.re.kr (Y.C.); jieun1008@add.re.kr (J.E.L.); jswon@add.re.kr (J.S.W.); manyounglee@add.re.kr (M.Y.L.); 2 Department of Mechanical Engineering, Ulsan National Institute of Science and Technology, Ulsan 44919, Republic of Korea; jinhobae@unist.ac.kr; 3 KAIST InnoCORE PRISM-AI Center, Korea Advanced Institute of Science and Technology, Daejeon 34141, Republic of Korea

**Keywords:** carbon fiber/silicon carbide (C/SiC) composites, chemical vapor infiltration (CVI), mechanical properties, molecular dynamics, micromechanics

## Abstract

A multiscale modeling approach is proposed to investigate the mechanical properties of carbon fiber/silicon carbide (C/SiC) composites fabricated by chemical vapor infiltration (CVI) process. First, reactive molecular dynamics simulations are conducted to estimate the mechanical properties of the SiC matrix fabricated via CVI. Subsequently, a two-level micromechanics-based homogenization is developed to account for the effects of various constituents (e.g., porosity and carbon fiber) on the mechanical properties of the C/SiC composites. A series of numerical parametric studies is performed to examine the influence of the model parameters on the mechanical properties of the C/SiC composites. In addition, experimental investigations, including tensile tests and scanning electron microscopy, are conducted to validate the proposed modeling approach. The results indicate that the proposed modeling approach provides predictions that are in good agreement with the experimental results, thereby demonstrating the effectiveness of the proposed modeling scheme.

## 1. Introduction

Ceramic matrix composite materials are key components of aircrafts, automobiles, and turbine engines [1,2]. These materials offer numerous advantages, including low density, oxidation resistance, thermal stability, and exceptional mechanical strength and stiffness [3,4]. Among the various ceramic matrix composites, carbon fiber/silicon carbide (C/SiC) composites have gained prominence as candidates because of their exceptional durability and resistance to heat and ablation, thus replacing more metal components [4,5,6,7,8]. Researchers have reported that C/SiC composites can maintain their mechanical performance at high-temperature conditions [9,10,11].

Generally, C/SiC composites are fabricated by introducing a SiC matrix into a carbon fiber preform using various infiltration methods such as melt infiltration, polymer infiltration and pyrolysis, and chemical vapor infiltration (CVI) [8,12,13,14,15]. Among them, the CVI method forms a SiC matrix on a carbon fiber preform using methyltrichlorosilane and a hydrogen catalyst at high temperature [16,17]. C/SiC composites fabricated by the CVI process can produce C/SiC composites with high fracture toughness and excellent thermodynamic properties, which can reduce the thermal damage at the fiber interface [16,17].

However, the effect of the CVI process on the mechanical properties of composites has not been clearly identified. CVI-processed C/SiC composites have inherent porosity because gas penetration is limited, and it is difficult to control the process parameters (e.g., temperature, pressure, gas flow, and mix ratio of gas) [16,17]. Another challenge with C/SiC composites is their varying mechanical performances, which stem from the inherent differences in scale between the SiC matrix and carbon fibers [18]. Therefore, accurate analyses and predictions of the mechanical properties of the composites are necessary. To address the inherent differences in analytical modeling, many researchers have suggested a multiscale model incorporating micromechanics and the finite element method to more precisely investigate the mechanical properties of CVI-processed C/SiC composites of varying scales [16,17,18,19,20]. Chateau et al. [19] developed a micromechanical model for predicting the mechanical properties of composites fabricated using a CVI process. Their model incorporated the effects of the porosity formed from the CVI process on the elastic properties of the composites. They reported that a small increase in the porosity volume fraction could potentially result in a drastic decrease in the transverse elastic properties at high stress concentrations [19]. Borkowski et al. [20] proposed a micromechanics-based multiscale model coupled with a thermoelastic progressive damage model to predict the elastic and damage behaviors of a plain-weave C/SiC composite system. They reported that the proposed multiscale model could account for the CVI-induced residual stresses and strains resulting in SiC matrix microcracking in C/SiC composites [20].

In addition, the carbon fiber preform in C/SiC composites is secured using a needle-punching process before the SiC matrix is introduced. Consequently, the carbon fibers in the composites are observed as chopped carbon fibers [17,18]. Therefore, the effect of the needle-punching process on the mechanical properties in composites is also significant. Researchers have suggested theoretical models using the finite element method and micromechanics to more precisely investigate the mechanical behaviors of the C/SiC composites with the needle-punched carbon fibers of varying scales [18,21]. Lim et al. [18] proposed an efficient multi-scale modeling approach to predicting the mechanical behavior of C/SiC composites with the needle-punched carbon fibers by means of a 3D high-fidelity finite element model integrated with computed tomography scanned images. Their modeling approach can simulate the force-displacement behavior of needle-punched C/SiC composites. Xie et al. [21] developed a micromechanical model to predict the nonlinear stress–strain behavior of the needle-punched C/SiC composites under tensile loading conditions. Their model incorporated the effects of SiC matrix cracking and debonding between the matrix and carbon fiber on the mechanical properties of the composites. Despite numerous studies predicting the mechanical behaviors of the needle-punched C/SiC composites using the CVI process, a comprehensive investigation that evaluates the significance of each constituent across various scales has yet to be performed.

To address this challenge, we propose a multiscale modeling approach to predict the mechanical properties of C/SiC composites fabricated using a CVI process. This study introduces reactive molecular dynamics (RMD) simulations to estimate the mechanical properties of a SiC matrix fabricated via CVI. This study adopts two-level homogenization incorporating a series of micromechanical models to predict the mechanical properties of C/SiC composites. Tensile tests are performed to examine the elastic behavior of the C/SiC tensile specimens fabricated using the CVI process. To evaluate the effectiveness of the modeling approach, the predicted mechanical properties of the C/SiC composites are compared with the experimental results. The agreement between the predictions and experimental results demonstrates the effectiveness of the proposed modeling scheme for simulating the mechanical properties of C/SiC composite materials.

## 2. Theoretical Background

**Figure 1:**
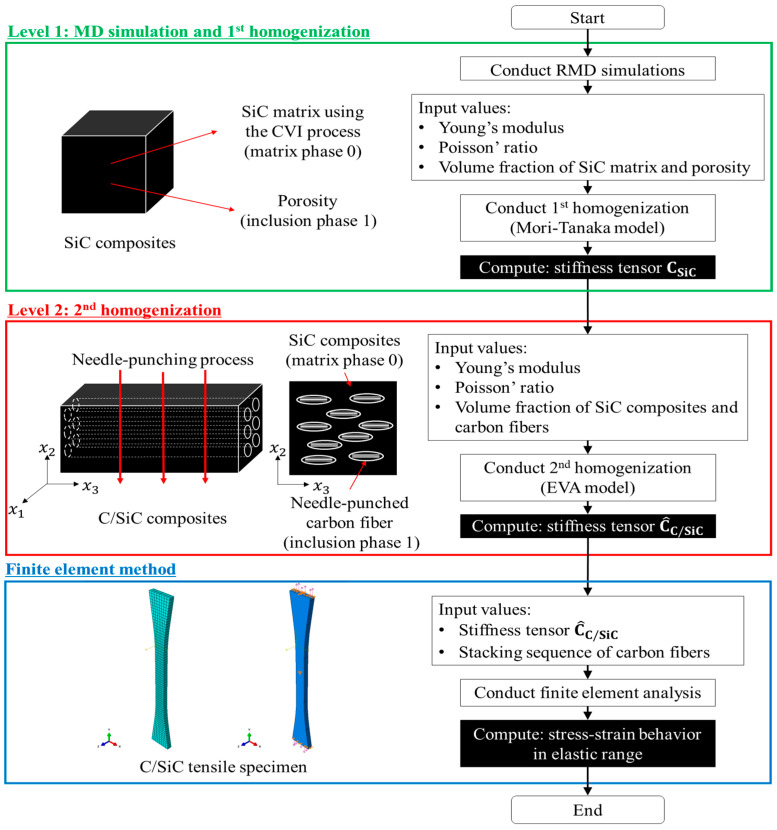
Flow diagram of the multiscale modeling approach for the prediction of the mechanical properties of C/SiC composites fabricated using a CVI process.

Figure 1 shows a flow diagram of the multiscale modeling approach for the prediction of the mechanical properties of C/SiC composites fabricated using the CVI process. This study employs RMD simulations and micromechanics-based homogenization to predict the mechanical properties of C/SiC composites across multiple length scales. At Level 1, RMD simulations are conducted to estimate the mechanical properties of the SiC matrix fabricated using the CVI process. The Mori-Tanaka micromechanical model is then adopted to estimate the mechanical properties of the SiC composites, which include the SiC matrix and inherent porosity for the 1st homogenization. At Level 2, the ensemble volume averaging (EVA) micromechanical model is utilized to determine the mechanical properties of the C/SiC composites with needle-punched carbon fibers for the 2nd homogenization. The resulting multiscale model is then incorporated into a finite element method to solve the boundary conditions of a C/SiC tensile specimen.

**Figure 2:**
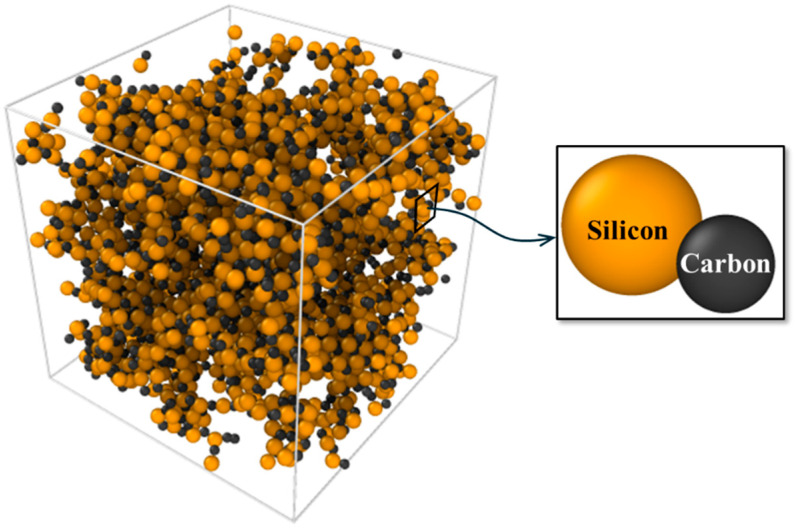
Molecular structures of a SiC molecular unit and initial configuration of the SiC matrix in a supercell.

Figure 2 shows the molecular structures of the SiC molecular unit and the initial configuration of the SiC matrix in a supercell visualized using OVITO (3.14.1) [22]. RMD simulations are conducted using a large-scale atomic/molecular massively parallel simulator (LAMMPS) to estimate the mechanical properties of the SiC matrix fabricated using a CVI process [23]. In this study, the reactive force field (ReaxFF) interatomic potential is adopted. This force field can be used empirically to investigate inter-atomic interactions on orders of atomic magnitude, considering their bonding, debonding, and rebonding, making it well-suited for modeling atomistic dynamics relevant to the CVI process [24,25]. However, it should be noted that the primary objective of these RMD simulations is to characterize the mechanical properties of the resulting amorphous SiC matrix rather than to simulate the deposition kinetics since the CVI process involves complex precursor chemistry (e.g., methyltrichlorosilane decomposition). We utilized the ReaxFF C/Si/H/O parameter set developed by Newsome et al. [26], which showed a good agreement with quantum mechanics data regarding the binding energy, equation of state, and heat of formation of crystalline SiC. This parameterization is therefore suitable for evaluating the Si–C bond dynamics during network stabilization and computing the elastic properties for the subsequent multiscale homogenization.

The initial atomistic configuration of a single SiC molecule has been constructed using the BIOVIA (v6.0) Materials Studio visualization module, a commercial molecular dynamics simulation package [27]. As shown in Figure 2, a cubic supercell with dimensions of 50 × 50 × 50 Å^3^ is modeled with periodic boundary conditions in all directions. This atomistic volume is intended to represent the intrinsic solid phase of the amorphous SiC matrix. It is noteworthy that the macroscopic porosity and fiber architecture, which exist on the micrometer scale, are far beyond the length scales used in present ReaxFF simulations. Therefore, these microstructural features are accounted for in the subsequent homogenization levels. An initial supercell representing amorphous SiC is generated from these molecules using PACKMOL (v0.1.13) [28], which builds geometrically optimized initial amorphous configurations for the RMD simulations. An initial matrix density of 1.5 g/cm^3^ is prescribed to represent an early-stage amorphous SiC matrix prior to its crystallization. Herein, the value of 1.5 g/cm^3^ is adopted based on preliminary simulations over a range of initial densities. This under-dense initial state facilitates progressive structural rearrangement during RMD simulations, thereby promoting an amorphous-to-more-ordered evolution toward a matrix density consistent with the experimentally observed density of the SiC matrix (2.16 g/cm^3^). The structure is subsequently infiltrated using RMD simulations to closely match the experimentally obtained density. The initial structure is then relaxed using the conjugate gradient minimization method with an energy tolerance of 1.0 × 10^−6^ kcal/mol and a force tolerance of 1.0 × 10^−8^ kcal/mol/Å, respectively. The structure is then equilibrated at 300 K via a canonical ensemble (NVT) simulation for 20 ps with a 0.2 fs timestep, using a Nosé-Hoover thermostat [29,30]. The model parameters and conditions used in the RMD simulations are listed in Table 1.

The mechanical properties of the simulated SiC matrix are determined using the strain-increment method. All simulations are performed at 300 K using an NVT ensemble equipped with a Nosé-Hoover thermostat [29]. Here, we use uniaxial compressive strain since tensile loading condition induces bond stretching, resulting in a rapid decrease in bond order; this can lead to premature bond transitions and the nucleation of localized voids within the amorphous network [31]. Uniaxial compressive strain is applied incrementally along the *x-*, *y-*, *z-* directions up to a maximum strain of ε = 0.1. The strain is iteratively increased at a rate of $\dot{\epsilon }$ = 1 × 10^−6^ fs^−1^ with a 0.2 fs timestep. Stress tensor components $\sigma_{ij}$ (where *i*, *j* = 1, 2, 3) calculated via the Virial theorem [32] are recorded at each strain increment after equilibration. The elastic modulus and Poisson’s ratio are then computed from the linear relationship between the applied strain components $\varepsilon_{kl}$ (*k* and *l* = 1, 2, 3) and the computed $\sigma_{ij}$ within this small strain regime [32].

After the RMD simulations on the SiC matrix, to estimate the mechanical properties of the SiC composites, including the SiC matrix and porosity, the Mori-Tanaka micromechanical model is adopted for 1st homogenization at Level 1. This Mori-Tanaka model considers the certain effects of inhomogeneity by considering the effective strain as the average strain in the matrix [33,34]. This model is generally used to estimate the elastic properties of composites with large volume fractions of inclusions [33]. Therefore, this model is applied at this level to achieve good accuracy and is computationally less intensive. In this modeling approach, two-phase composites are considered: a SiC matrix (matrix phase 0) and porosity (inclusion phase 1). The present modeling approach assumes that all effective inclusions are randomly oriented with perfect interface conditions and spherical shapes. Further details regarding the Eshelby tensor are available in the literature [33,34]. However, it should be noted that the present modeling approach assumes spherical porosity without the non-spherical porosity or coalescence in the SiC composites. Accordingly, the present modeling approach cannot consider the coalescence of porosity.

To account for the effect of needle-punching on the mechanical properties of C/SiC composites, the present modeling approach assumes that needle-punched carbon fibers are represented as ellipsoidal inclusions, as shown in Figure 1. At 2nd homogenization at Level 2, the EVA micromechanical model, which is a micromechanics-based model proposed by Ju and Chen [35], is applied to estimate the mechanical properties of the C/SiC composites with needle-punched carbon fibers. This EVA model can account for the effects of various inclusions within the matrix on the effective moduli of elastic composites containing randomly dispersed ellipsoidal inhomogeneities, which makes it particularly useful for this stage of analysis [33,35].

Therefore, let us consider a two-phase composite consisting of the SiC composites (matrix phase 0) and ellipsoidal carbon fiber inclusions (inclusion phase 1). Using the EVA micromechanics-based model, the stiffness tensor for two-phase composites, denoted as $C_{C/SiC}$, is given by Equation (1) as follows [36,37,38]:

(1) $$C_{C/SiC}=C_{SiC}\cdot \left[I+\;\sum_{n=1}^{2}\left[\phi_{n}{\left(A_{n}+S_{n}\right)}^{-1}\cdot {\left\{I-\phi_{n}S_{n}\cdot {\left(A_{n}+S_{n}\right)}^{-1}\right\}}^{-1}\right]\right],$$

where I and $C_{SiC}$ are the identity and fourth-rank stiffness tensors, respectively, for the SiC composites. Note that the value of $C_{SiC}$ is determined by substituting the homogenized results estimated using the Mori-Tanaka micromechanical model at Level 1.$\phi_{n}$, $S_{n}$ and $A_{n}$ are the volume fraction, the Eshelby’s tensor, and the strain concentration tensor of an *n*-phase inclusion, respectively [36,37,38]. Here, the aspect ratio of the ellipsoidal carbon fiber inclusions $\alpha_{C}$ is calculated as $\alpha_{C}=L_{C}/R_{C}$, using the material properties of carbon fibers specified in Section 4.2, where $R_{C}$ and $L_{C}$ are the radii of the prolate spheroid along the minor and major axes, respectively [38].

Furthermore, in this modeling approach, the 3D orientational averaging method proposed by Lee and Simunovic [39] is applied to the stiffness tensor to consider the effect of randomly oriented or unidirectionally aligned carbon fiber inclusions on the mechanical properties of the composites [38,39]. The 3D orientational averaged stiffness tensor can be expressed using Equation (2), as follows [33,37,38]:

(2) $${\hat{C}}_{C/SiC}={\hat{C}}_{IK}^{\left(1\right)}\delta_{ij}\delta_{kl}+{\hat{C}}_{IJ}^{\left(2\right)}\left(\delta_{ik}\delta_{jl}+\delta_{il}\delta_{jk}\right),$$

where the components of the explicit tensors ${\hat{C}}_{IK}^{\left(1\right)}$ and ${\hat{C}}_{IJ}^{\left(2\right)}$ are functions of $C_{IK}^{\left(1\right)}$ and $C_{IJ}^{\left(2\right)}$ from Equation (1); its components can be found in references [33,39]. Note that the hat indicates the values after the orientational averaging process.

## 3. Parametric Studies

### 3.1. RMD Simulation Results

**Figure 3:**
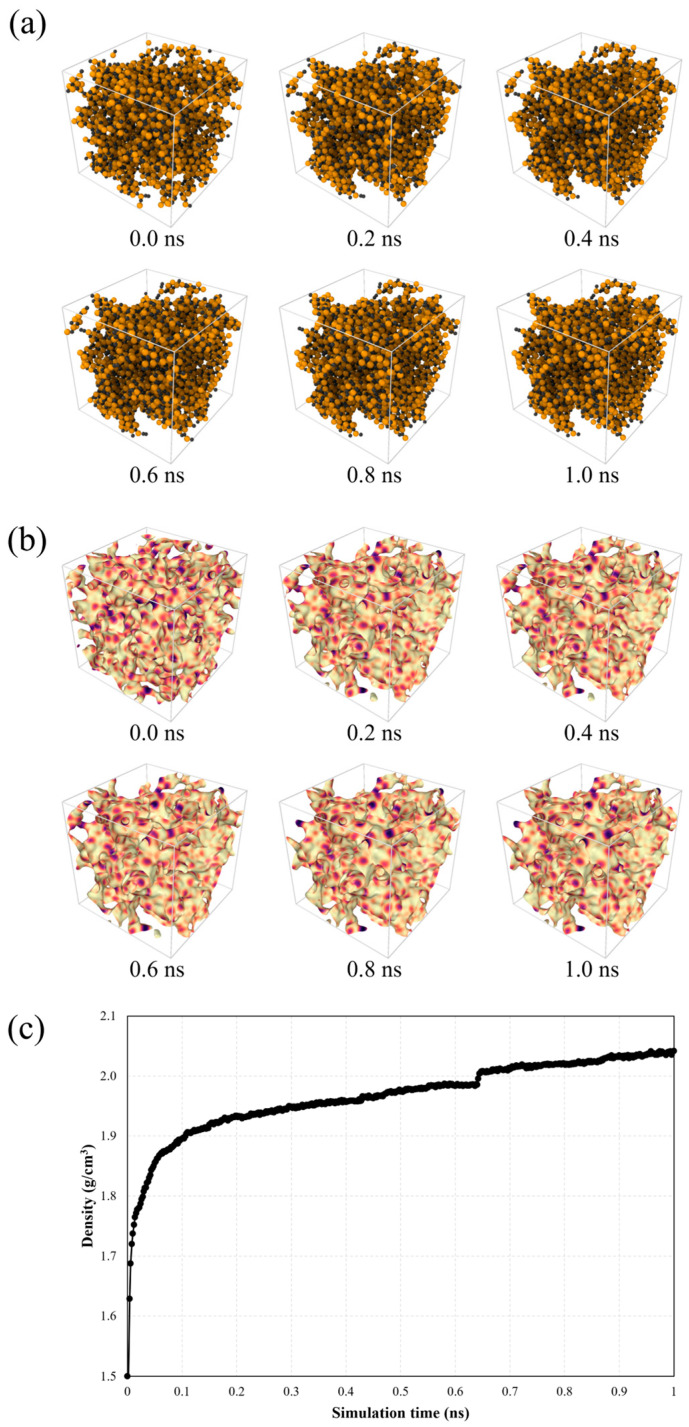
RMD simulation results of the SiC matrix fabricated using a CVI process at initial configuration and at 0.2, 0.4, 0.6, 0.8, and 1.0 ns with (**a**) atomic configurations and (**b**) volumetric morphologies as well as (**c**) progressive changes in true density of SiC matrix. The color representations used in this figure denote as follows: C-black, Si-orange, high-density SiC agglomerates-yellow/white, and lower packing and porosity-purple/black.

Figure 3 shows the RMD simulation results of the SiC matrix fabricated using the CVI process at the initial configuration and at 0.2, 0.4, 0.6, 0.8, and 1.0 ns with atomic configurations and volumetric morphologies, as well as the progressive changes in the true density of the SiC matrix. As shown in Figure 3a, the Si and C atoms are initially sparsely distributed. However, as the RMD simulations proceed, they undergo significant rearrangement, which is an interconnected structure characteristic of amorphous or nanocrystalline SiC, resulting in the formation of a denser structure. In the volumetric morphologies shown in Figure 3b, a uniform sequential color map (magma) is applied, with the color intensity corresponding to local mass density variations. Volumetric representations further highlight the changes in atomic density and internal morphology, demonstrating a transition from a highly porous structure to a denser and more uniform SiC matrix. Figure 3c shows that this structural development is most pronounced within the first 0.2 ns reaching, after which the atomic network becomes increasingly stabilized, showing that the true density reaches approximately 2.04 g/cm^3^ at 1.0 ns of the RMD simulations.

**Figure 4:**
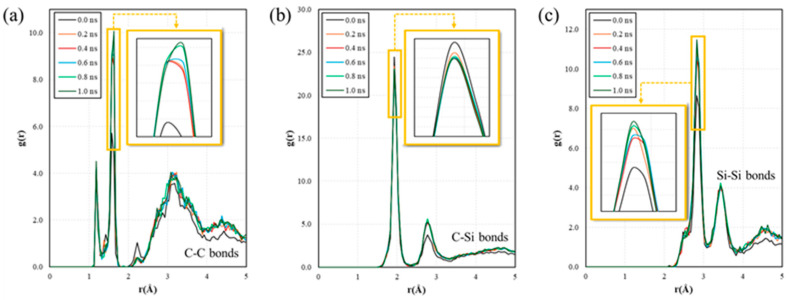
Radial distribution functions of (**a**) C-C, (**b**) C-Si, and (**c**) Si-Si atomic pairs at different simulation times of simulated SiC matrix, with an enlargement of specific range.

The structural evolution of the simulated SiC matrix is further analyzed using radial distribution functions (RDFs). Figure 4 shows the RDFs of the C-C, C-Si, and Si-Si atomic pairs at different RMD simulation times for the simulated SiC matrix with an enlargement of the specific range. As shown in Figure 4a, the C-C RDF peak (r ≈ 1.5 Å) significantly sharpens over the RMD simulation time, indicating C-C bond formation and increased local medium-range order. Concurrently, the dominant C-Si peak (r ≈ 1.9 Å) intensifies and narrows, reflecting the rapid establishment and optimization of the primary Si-C network framework in Figure 4b. The Si-Si RDF peak (r ≈ 3 Å), characteristic of Si-C-Si bonds becomes more defined, signifying growing medium-range order as shown in Figure 4c. Consequently, this tracing RDF trend illustrates the transition from an initially disordered atomic arrangement to an increasingly ordered SiC network through a progressive atomic rearrangement during the simulation.

**Figure 5:**
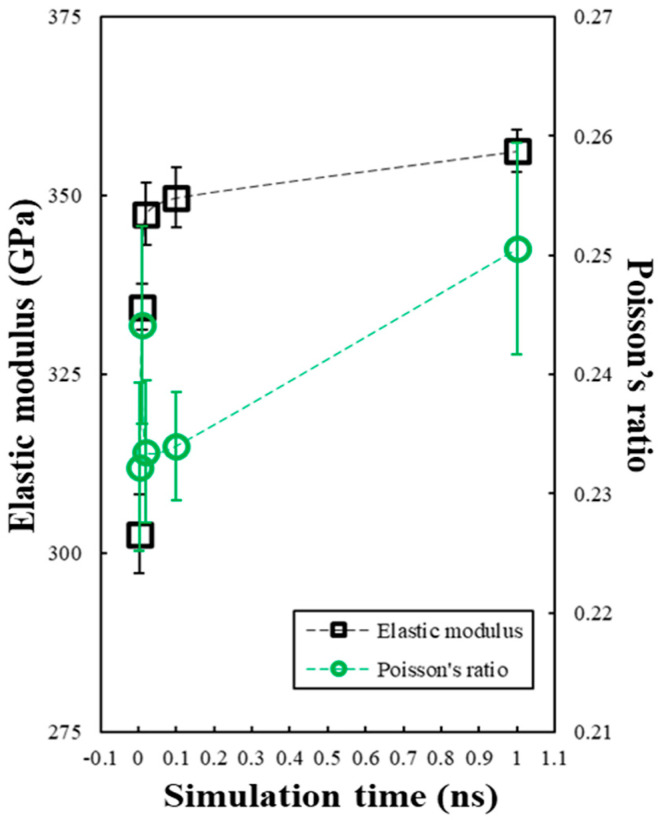
The elastic modulus and Poisson’s ratio of the simulated SiC matrix over the simulation time.

Figure 5 shows the elastic modulus and Poisson’s ratio of the simulated SiC matrix over time. The elastic modulus (black squares, left axis) and Poisson’s ratio (green circles, right axis) are determined at various stages of structural development from 0.0 to 1.0 ns. The elastic modulus exhibits a pronounced initial increase, rising from 302.73 ± 5.46 GPa at 0.0 ns to 349.75 ± 4.14 GPa by 0.1 ns. Thereafter, the growth rate decreases with the modulus stabilizing at 356.27 ± 2.90 GPa at 1.0 ns. Poisson’s ratio follows a similar trend, increasing from 0.232 ± 0.0070 to 0.234 ± 0.0045 within the first 0.1 ns, and gradually reaching 0.251 ± 0.0089 at 1.0 ns. These results reflect the rapid densification and structural rearrangement of the SiC matrix during the early stages of the RMD simulations, followed by progressive stabilization of the mechanical properties.

### 3.2. Effects of the Volume Fraction of Porosity on the Elastic Modulus of the SiC Composites

**Figure 6:**
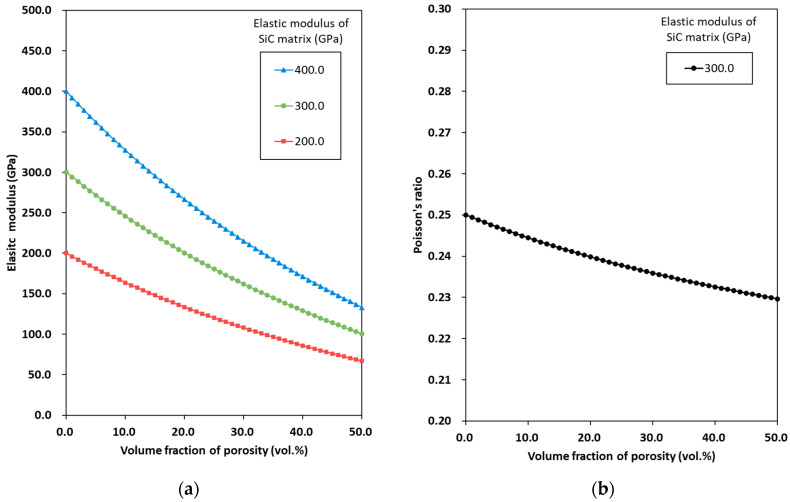
Predicted elastic modulus of C/SiC composites with varying (**a**) aspect ratios of the carbon fiber and (**b**) volume fractions of the carbon fiber for both aligned and random orientations.

A series of parametric studies is performed to assess the sensitivity of the proposed multiscale modeling approach to variations in the mechanical properties of the C/SiC composites. The effects of the volume fraction of porosity on the elastic modulus of the SiC composites at 1st homogenization at Level 1 are investigated. Figure 6 shows the predicted elastic modulus $E_{SiC}$ and Poisson’s ratio $\nu_{SiC}$ of SiC composites with varying contents of porosity. The elastic modulus of the SiC matrix changes from 200 to 400 GPa, whereas Poisson’s ratio remains constant at 0.25, based on the RMD simulation results. As shown in Figure 6a, the value of $E_{SiC}$ value decreases significantly as the porosity increases. The effect of porosity on $E_{SiC}$ becomes noticeable as the value of the elastic modulus of the SiC matrix increases. Meanwhile, Figure 6b shows the effect of porosity on $\nu_{SiC}$. For the case of $E_{SiC}$ = 300 GPa, the value of $\nu_{SiC}$ slightly decreases as the value of porosity increases. It is evident that porosity significantly affects the mechanical properties of SiC composites [16,19].

### 3.3. Effects of the Carbon Fiber Geometry and Orientation on the Elastic Modulus of the C/SiC Composites

A parametric study of the C/SiC composites at 2nd homogenization at Level 2 is subsequently discussed. These parametric studies explore the effects of carbon fiber geometry and orientation on the elastic modulus of the C/SiC composites. The parameters used in the parametric simulations of the C/SiC composites are listed in Table 2. Here, the value of $E_{SiC}$ is assumed to be 100 GPa based on the parametric study in Section 3.2 and the experimentally measured volume fraction of porosity of specimens in Section 4.2.

**Figure 7:**
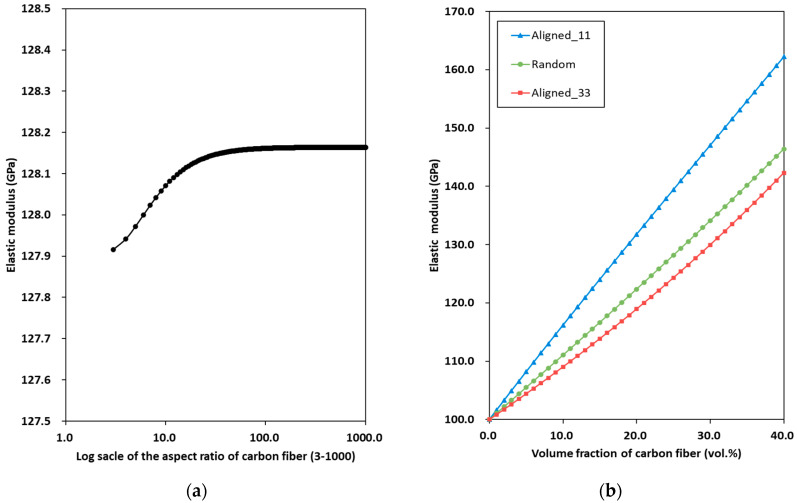
Predicted elastic modulus of C/SiC composites with varying (**a**) aspect ratios of the carbon fiber and (**b**) volume fractions of the carbon fiber for both aligned and random orientations.

Figure 7 shows the predicted elastic modulus of C/SiC composites $E_{C/SiC}$ with varying aspect ratios of the carbon fiber $\alpha_{C}$ and volume fractions of the carbon fiber $\phi_{2}$ for both aligned and random orientations. Figure 7a shows $E_{C/SiC}$ with varying $\alpha_{C}$. The carbon fibers are assumed to have random orientations. Two distinct trends emerge. In the first range, with $\alpha_{C}$ varying from 3 to 100, $E_{C/SiC}$ increases noticeably as $\alpha_{C}$ approaches 100. In the second range, from 100 to 1000, $E_{C/SiC}$ remains constant. These results suggest that a lower aspect ratio of carbon fibers is expected to generate a lower stiffness in C/SiC composites [38]. Figure 7b shows $E_{C/SiC}$ with varying $\phi_{2}$ for both aligned and random orientations. Note that Aligned_11 and Aligned_33 represent cases with unidirectionally aligned carbon fiber inclusions oriented along the longitudinal and transverse directions, respectively. The $E_{C/SiC}$ ratio for the Aligned_11 case is higher than that for the Random and Aligned_33 cases. These results suggest that the orientation of the carbon fibers considerably influences the mechanical properties of the C/SiC composites, as reported in the literature [33].

## 4. Model Verification

### 4.1. Specimen Preparation and Test Methods

**Figure 8:**
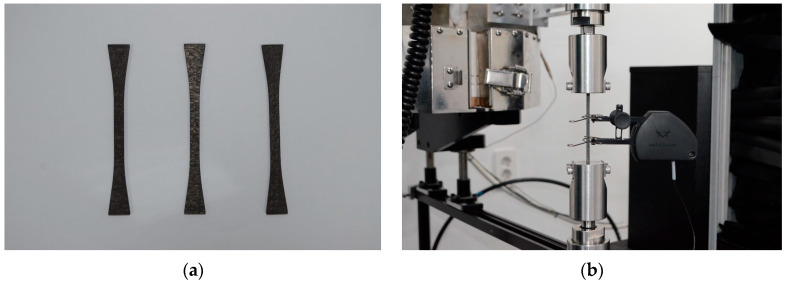
(**a**) C/SiC tensile specimens and (**b**) tensile test.

A C/SiC tensile specimen was fabricated, and a tensile test was performed. Note that the present study fabricated a [0/90/0/90] C/SiC tensile specimen for model verification; “0” denotes the ply oriented parallel to the tensile loading direction, whereas “90” signifies the ply oriented perpendicular to the loading direction. Figure 8 shows the tensile C/SiC specimens and the tensile test. A carbon fiber preform composed of a 25.0 vol.% unidirectional fiber was used. The unit layers were stacked alternately in the perpendicular directions (0° and 90°). A needle-punching process was applied to adhere the adjacent plies after each layer was added. The fabrication process for the CVI-processed C/SiC composite specimens was similar to that described in a previous study [40]. The SiC matrix was formed through a CVI process using methyltrichlorosilane, resulting in C/SiC composites. All dimensions of the tensile specimen were determined with reference to ASTM C1275-18 [41]. In particular, the detailed dimensions can be found in the ASTM standard [41]. Subsequently, C/SiC tensile specimens with a thickness of 3.0 mm and a length of 111.7 mm were machined to evaluate their mechanical properties, as shown in Figure 8a.

To verify the proposed modeling approach, the elastic behavior of the C/SiC tensile specimen was evaluated using a universal testing machine (INSTRON 5882, Instron Corporation, Norwood, MA, USA) in accordance with ASTM standard C1275-18 [41]. The universal testing machine has a maximum crosshead speed of 1.0 mm/min and a maximum loading capacity of ten tons. The strain rate was maintained at 0.0001 s^−1^ under quasi-static tensile loading conditions. Strain measurements were obtained using a linear variable-displacement transformer extensometer with a gauge length of 25.0 mm, as shown in Figure 8b. The representative elastic modulus was obtained by averaging the results of three replicate specimens. After the tensile test, the morphological characteristics of the specimens were examined using scanning electron microscopy (SEM, FEI Quanta 650, FEI Company, Hillsboro, OR, USA).

### 4.2. Experimental Results

**Figure 9:**
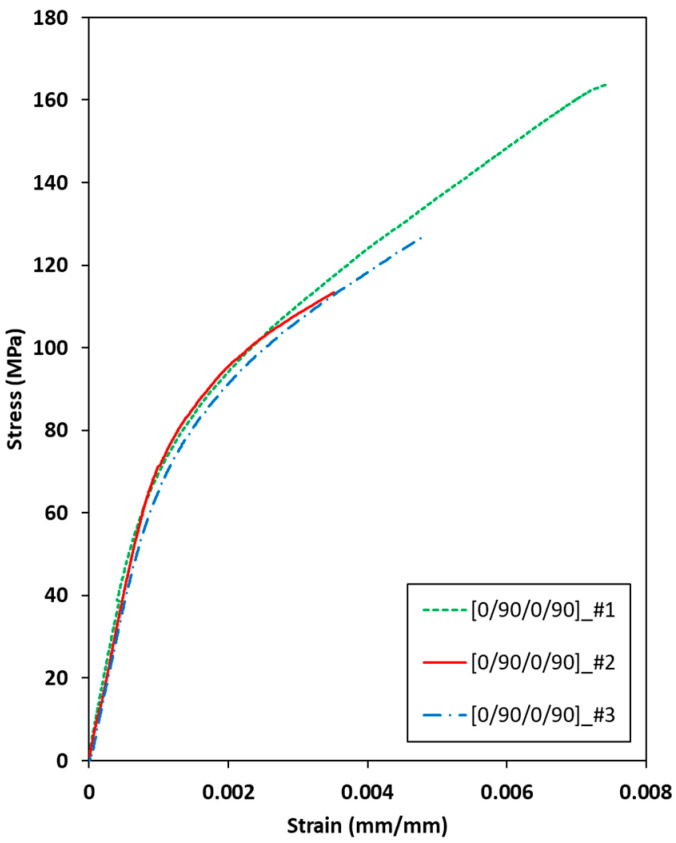
Measured stress–strain behaviors of the [0/90/0/90] C/SiC tensile specimen under uniaxial tension.

Figure 9 shows the measured stress–strain behavior of the [0/90/0/90] C/SiC tensile specimen under uniaxial tension. The density of all specimens averaged 2.16 g/cm^3^, with a negligible standard deviation of less than 0.0003 g/cm^3^, and the volume fraction of porosity of all specimens was estimated to 46.7 vol.%. The ultimate tensile strength of all the specimens exceeded 100.0 MPa. The stress values for all C/SiC tensile specimens increased linearly from 0 to 0.001 strain. The elastic modulus was estimated as 86.55 GPa ([0/90/0/90]_#1), 79.29 GPa ([0/90/0/90]_#2), and 83.88 GPa ([0/90/0/90]_#3), with a small standard deviation of 4.14. Generally, the elastic modulus of C/SiC composites ranges from 50.0 to 90.0 GPa at room temperature [11]. Therefore, the results suggest that the present C/SiC tensile specimens were uniformly fabricated.

**Figure 10:**
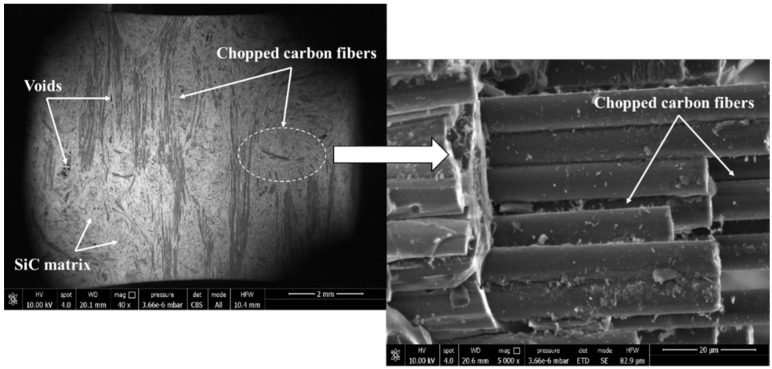
SEM images of the morphology and structure of the [0/90/0/90] C/SiC tensile specimen (Scale bars indicate 2 mm (**left**) and 20 μm (**right**), respectively).

The SEM images of the morphology and structure of the [0/90/0/90] C/SiC tensile specimens are shown in Figure 10. The carbon fibers were surrounded by a SiC matrix and interspersed with porosity, and carbon fibers were cut within the SiC matrix. The magnified SEM image showed the chopped carbon fibers resulting from the needle-punching process in the C/SiC composites [17,18]. Furthermore, it was observed that the chopped carbon fibers are unidirectionally aligned in the matrix. Therefore, it is assumed that the chopped carbon fibers are unidirectionally aligned in the longitudinal direction (Aligned_11) in the present prediction. The representative length and diameter of the chopped carbon fibers were obtained by averaging the measurements from ten chopped carbon fibers in the SEM images. The measured length and diameter of the chopped carbon fibers are listed in Table 3. The average length and diameter of the fibers were measured as 2.50 mm and 0.15 μm, respectively. The standard deviation of the measured length and diameter of the fibers were 0.82 and 0.15, respectively. Therefore, the calculated value of the aspect ratio (353.0) by dividing the average length by the average diameter of the chopped carbon fibers was used in the present prediction.

## 5. The Comparison Between the Present Prediction and Experimental Results

The predicted elastic behavior of the C/SiC tensile specimen is compared with the experimental results. For the [0/90/0/90] C/SiC tensile specimen, the following material properties are utilized: $E_{C}$ = 100.0 GPa [42], $\nu_{C}$ = 0.20, $R_{C}$ = 7.0, $\alpha_{C}$ = 353.0, $\phi_{1}$ = 75.0 vol.%, and $\phi_{2}$ = 25.0 vol.%. Note that the RMD simulation results at 1.0 ns ($E_{SiC}$ = 356.27 GPa and $\nu_{SiC}$ = 0.25 at 1.0 ns) in Section 3 are utilized in these predictions for incorporating the CVI-processed effect. All other model parameters and methods used for the predictions are consistent with those outlined in Section 4.1 and Sectio 4.2.

**Figure 11:**
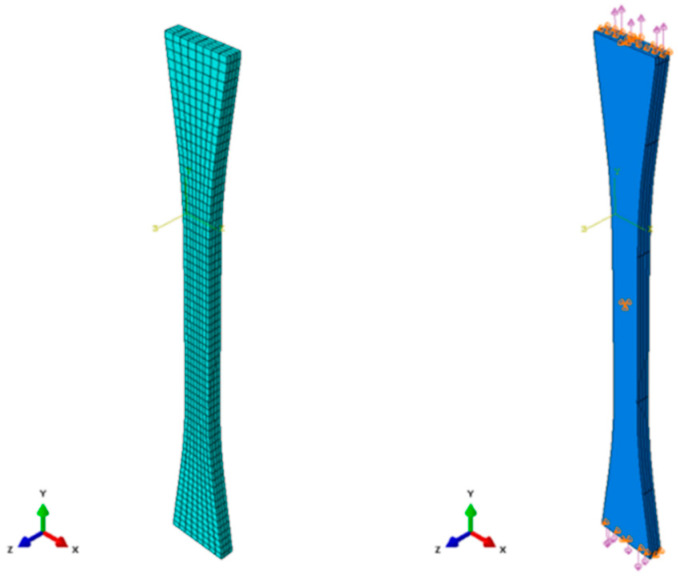
The meshes, loads, and constraints applied to the [0/90/0/90] C/SiC tensile specimen. The color representations used in this figure denote as follows: load direction-purple, boundary condition-orange, and *x*-, *y*-, *z*- directions-yellow.

Subsequently, a finite element analysis is conducted to simulate the elastic behavior of the [0/90/0/90] C/SiC tensile specimen, considering the shape of the tensile specimen and the stacking sequence of carbon fibers. The proposed multi-level homogenization modeling approach is implemented in the finite element code ABAQUS to solve the boundary conditions of the specimen under tensile loading. Commercially available software ABAQUS (ABAQUS/CAE 2023 version) is used to model the specimen. Unidirectionally aligned carbon fibers in the longitudinal direction (Aligned_11) are assumed to consider the needle-punching effect of the carbon fiber preform. Then, the calculated stiffness matrix ${\hat{C}}_{C/SiC}$ obtained from the proposed modeling approach is applied to each ply. The meshes, loads, and constraints applied to the [0/90/0/90] C/SiC tensile specimens are shown in Figure 11. Details, including the dimensions of the tensile specimen, are provided in ASTM C1275-18 [41]. The modeled C/SiC tensile specimen consists of four plies, each 0.75 mm thick, as detailed in Section 4.1. The plies are stacked with 0° and 90° rotation angles, and perfect bonding is assumed between each ply, with the interfacial area maintained until the specimen reaches its peak strength. The modeled specimen comprises 2112 elements, all of which are 3D eight-node (C3D8) linear brick solid elements. As shown in Figure 11, the displacements of both specimen edges in the *z*-direction and the central surface of the specimen in all directions are constrained. A virtual load of 1500 N is applied to each edge in the longitudinal direction (*y*-direction) to simulate the elastic behavior of the specimen. The stress–strain data are obtained from the point of the central surface, which is the identical point of the attached gauge.

**Figure 12:**
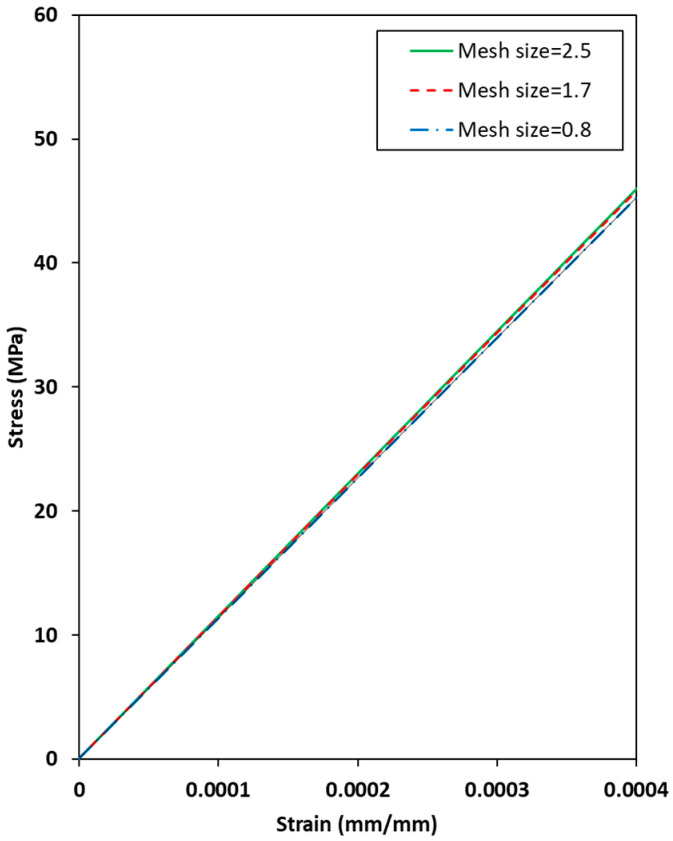
Simulated elastic behaviors of the [0/90/0/90] C/SiC tensile specimens to different global mesh sizes.

Figure 12 shows the simulated elastic behaviors of the [0/90/0/90] C/SiC tensile specimens with different global mesh sizes. The number of elements of the 2.5, 1.7, and 0.8 global mesh sizes, which are composed of the elastic behaviors, is 1104, 2112, and 10,224, respectively, in the range of a displacement of 1–0.0004 strain. The stress values of all mesh sizes converged to approximately 45 MPa at a strain of 0.0004, although the load value of the 2.5 global mesh size is slightly higher than those of the other mesh sizes owing to an insufficient dataset. Therefore, the mesh size in the present simulations is determined to be 1.7 global mesh size based on the results shown in Figure 12 due to the computational limitations in considering a huge number of elements in the 0.8 global mesh size.

**Figure 13:**
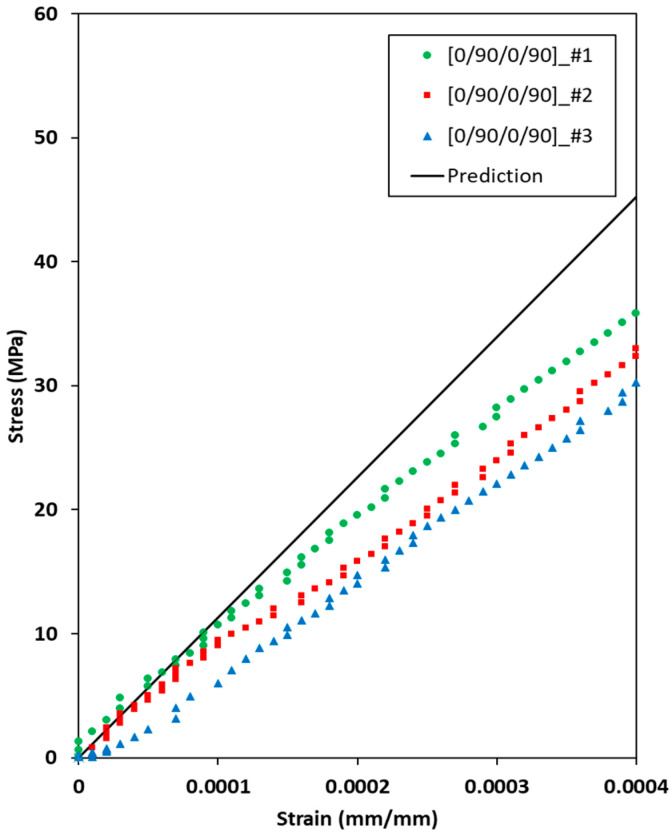
Comparisons of the elastic behavior of the [0/90/0/90] C/SiC tensile specimen under uniaxial tension between the present prediction and experimental results.

Figure 13 compares the elastic behavior of the [0/90/0/90] C/SiC tensile specimens under uniaxial tension between the present prediction and experimental results. The predicted elastic behavior of the [0/90/0/90] C/SiC tensile specimen shows fair agreement with the experimental results in the strain range of 0–0.0001. However, the predicted stress value of the [0/90/0/90] C/SiC tensile specimens is shown to be higher than the experimental results after 0.0001 strain. The assumptions that the unidirectionally aligned carbon fibers in the longitudinal direction are thoroughly homogenized in the C/SiC composites and the deterioration of carbon fibers in the composites by high-temperature environments in the CVI process are attributable to the difference between the present predictions and experimental results [9,18,42].

## 6. Concluding Remarks

A multiscale modeling approach for predicting the mechanical properties of C/SiC composites fabricated by the CVI process was proposed. RMD simulations were conducted to estimate the mechanical properties of the SiC matrix, which is fabricated by the process of CVI. Two-level micromechanics-based homogenization was introduced to estimate the effects of the volume fractions of porosity and carbon fibers. The proposed multiscale modeling approach was then implemented in a finite element code to simulate the elastic behavior of the C/SiC tensile specimen. In addition, experimental investigations were conducted on C/SiC tensile specimens to validate the assumptions and predictions of the proposed multiscale model. The findings of this study can be summarized as follows:

(1) The RMD simulation results for the SiC matrix fabricated using the CVI process were used to predict the mechanical properties of the C/SiC composites. The predicted elastic behavior of the C/SiC composites yielded the final results of the mechanical properties of the composites, which were within a reasonable range of the experimental results.
(2) In the parametric studies, the volume fractions of porosity significantly affected the mechanical properties of the SiC composites, notably reducing the elastic modulus as their volume fractions increased. Meanwhile, a higher aspect ratio of the carbon fiber inclusions, particularly those aligned in the longitudinal direction, yielded higher stiffness in the C/SiC composites.
(3) C/SiC tensile specimens were fabricated using the CVI process, and tensile tests were conducted. The average elastic modulus of the specimen and aspect ratio of chopped carbon fibers were measured as 83.24 GPa and 353.0, respectively.

This study demonstrates the effectiveness of a multiscale modeling approach for determining the mechanical properties of C/SiC composites. It is expected that the proposed multiscale modeling approach can be used to investigate the effective mechanical properties of C/SiC composites and design the needle-punched C/SiC composites. However, as reported in the literature, C/SiC composites are complex materials whose properties are significantly affected by their constituents. The assumptions made in this study can influence the final predictive capability of the present modeling approach. To assess the application of this approach more accurately, a supplementary model verification of C/SiC composites with varying volume fractions of their constituents should be conducted. Furthermore, it should be noted that relevant parameters capable of considering other physical properties of materials (i.e., the microstructure, coalescence, and fiber/matrix interface damage) in the present modeling approach are needed for more accurate and realistic predictions. The proposed modeling approach will be extended to include the study of progressive damage behavior in C/SiC composites and to integrate larger-scale simulation methods such as kinetic Monte Carlo to simulate the deposition profiles and pore evolution during the CVI process.

## Figures and Tables

**Table 1:** Model parameters and conditions used in the ReaxFF-based RMD simulations.

Parameter and Condition	Present Simulation
Forcefield	ReaxFF
Parameters in forcefield	C/Si/H/O ^1^
RMD ensemble simulation	NVT
Temperature (K)	300
Temperature control	Nose-Hoover thermostat
Energy minimization	Conjugate gradient algorithm
Unit cell size (Å^3^)	50 × 50 × 50
Total number of atoms	5632

^1^ Note that the parameters of the C/Si/H/O datasets are determined by referring to Newsome et al. [26].

**Table 2:** Parameters used in the parametric simulations for C/SiC composites.

Parameter	Present Simulation
Elastic modulus of SiC composites $E_{SiC}$ (GPa)	100.0
Poisson’s ratio of SiC composites $\nu_{SiC}$	0.25
Elastic modulus of carbon fiber ^1^ $E_{C}$ (GPa)	240.0
Poisson’s ratio of carbon fiber ^1^ $\nu_{C}$	0.20
Radius of carbon fiber $R_{C}$ (μm)	7.0
Aspect ratio of carbon fiber $\alpha_{C}$	300.0
Volume fraction of SiC composites $\phi_{1}$ (vol.%)	75.0
Volume fraction of carbon fiber $\phi_{2}$ (vol.%)	25.0

^1^ Note that the values of the parameters are determined by referring to literature values [37,38].

**Table 3:** Measured length and diameter of the chopped carbon fibers.

No.	Length (mm)	Diameter (μm)
1	3.19	7.00
2	2.82	6.90
3	2.54	7.07
4	1.31	7.00
5	2.11	7.12
6	2.56	7.47
7	1.73	7.00
8	1.72	7.22
9	4.33	7.00
10	2.68	7.09
Average	2.50	7.09
Standard deviation	0.82	0.15

## Data Availability

The original contributions presented in this study are included in the article. Further inquiries can be directed to the corresponding author.

## Abbreviations

The following abbreviations are used in this manuscript:

C/SiC	Carbon fiber/silicon carbide
CVI	Chemical vapor infiltration
RMD	Reactive molecular dynamics
EVA	Ensemble volume averaging
LAMMPS	Large-scale atomic/molecular massively parallel simulator
ReaxFF	Reactive force field
RDFs	Radial distribution functions
